# Long-term effects of smallpox vaccination on expression of the HIV-1 co-receptor CCR5 in women

**DOI:** 10.1371/journal.pone.0207259

**Published:** 2018-11-15

**Authors:** K. B. Beck, B. L. Hønge, J. S. Olesen, M. S. Petersen, S. Jespersen, C. Wejse, Z. J. da Silva, C. Medina, D. D. S. Té, B. K. Moeller, C. S. Benn, P. Aaby, C. Erikstrup

**Affiliations:** 1 Bandim Health Project, Indepth network, Bissau, Guinea-Bissau; 2 Department of Clinical Immunology, Aarhus University Hospital, Aarhus, Denmark; 3 Department of Infectious Diseases, Aarhus University Hospital, Aarhus, Denmark; 4 GloHAU, Center for Global Health, Dept of Public Health, Aarhus University, Aarhus, Denmark; 5 National HIV programme, Ministry of Health, Bissau, Guinea-Bissau; 6 Research Center for Vitamins and Vaccines (CVIVA), Bandim Health Project, Statens Serum Institut, Copenhagen, Denmark; University of Cape Town, SOUTH AFRICA

## Abstract

**Background:**

Smallpox vaccinations were stopped globally in 1980. Recent studies have shown that in women, being smallpox vaccinated was associated with a reduced risk of HIV infection compared with not being smallpox vaccinated. At the initial infection, HIV-1 most often uses CCR5 as a co-receptor to infect the T-lymphocytes. We therefore investigated whether smallpox vaccination is associated with a down-regulation of CCR5 on the surface of peripheral T-lymphocytes in healthy women in Guinea-Bissau.

**Methods:**

We included HIV seronegative women from Bissau, Guinea-Bissau, born before 1974, with and without a smallpox vaccination scar. Blood samples were stabilised in a TransFix buffer solution and stained for flow cytometry according to a T-cell maturation profile.

**Results:**

Ninety-seven women were included in the study; 52 with a smallpox vaccination scar and 45 without a scar. No association between smallpox vaccination scar and CCR5 expression was found in any T-lymphocyte subtype.

**Conclusion:**

Among HIV seronegative women, being smallpox vaccinated more than 40 years ago was not associated with a down-regulation of CCR5 receptors on the surface of peripheral T-lymphocytes.

## Introduction

HIV-1 replication is initiated by binding of the glycoproteins gp120 and gp41 to the primary receptor CD4, and a co-receptor. During the initial infection, HIV-1 most often uses CCR5 as the co-receptor. Therefore, individuals with a 32-nucleotide deletion (Δ32) within the CCR5-gene, resulting in an inactive receptor, are largely protected from HIV-1 infection [1]. The critical role of CCR5 is illustrated in the case report of Timothy Ray Brown, known as the *Berlin patient*, who is the only individual ever cured of HIV [2, 3]. He was diagnosed with acute leukaemia and was treated with allogeneic stem cell transplantation from a donor homozygous for Δ32. This resulted in complete remission of his leukaemia disease as well as eradication of the HIV infection.

Vaccinia vaccination has been used as a vaccination against smallpox, as vaccinia virus confers cross-protection against smallpox. The last case of naturally occurring smallpox was recorded in 1977 [4], and in 1980, vaccination against smallpox was stopped globally. Smallpox vaccination is associated with a strong immune response [5] and may have non-specific effects that cannot be explained by the prevention of smallpox [6]. An association between smallpox vaccination and a reduced risk of mortality and hospitalisation among adults has been found in Guinea-Bissau [7, 8] and in Denmark [9, 10].

In addition, studies from Guinea-Bissau and Denmark show that individuals vaccinated against smallpox and/or Bacille-Calmette-Guérin (BCG) had a reduced risk of HIV-1 infection compared with non-vaccinated individuals [11]. This effect tended to be stronger in women, suggesting a gender-specific effect as seen for other vaccines. In Guinea-Bissau, the protective effect was mainly found among individuals with two or more smallpox vaccination scars [11].

The biological mechanism behind the possible protective effect of smallpox vaccination against HIV-1 in women is unknown, but not biologically implausible. First, down-regulation of CCR5 from the surface of T-cells infected by smallpox vaccination can be demonstrated *ex vivo* [12]. Secondly, an up to five-fold reduction in CCR5-tropic HIV-1 replication in peripheral blood mononuclear cells (PBMCs) cultivated *in vitro* from vaccinia vaccinated individuals compared with unvaccinated individuals is reported after 3–6 months of follow-up [13]. Hypothetically, smallpox vaccination could thus decrease HIV replication through down-regulation of CCR5 on peripheral blood mononuclear cells (PBMCs).

The aim of this study was to compare the CCR5-expression on peripheral T-lymphocytes between HIV-uninfected women with and without a scar from smallpox vaccination. The women were vaccinated before smallpox vaccination was stopped more than 35 years ago. Hence, we were only able to study the long-term effects of smallpox vaccination on CCR5-expression among women who had survived to be enrolled in the study.

## Methods

### Setting and study population

The study was a case-control study. HIV-seronegative Guinean women with and without a scar from smallpox vaccination were included.

From November 2014 to February 2016, an HIV prevalence survey was conducted within the Bandim Health Project, a Health and Demographic Surveillance System Site in Guinea-Bissau [14]. A total of 2,603 individuals were included in the three suburbs of Bissau, hereof 1,504 (57.8%) were women.

**Fig 1:**
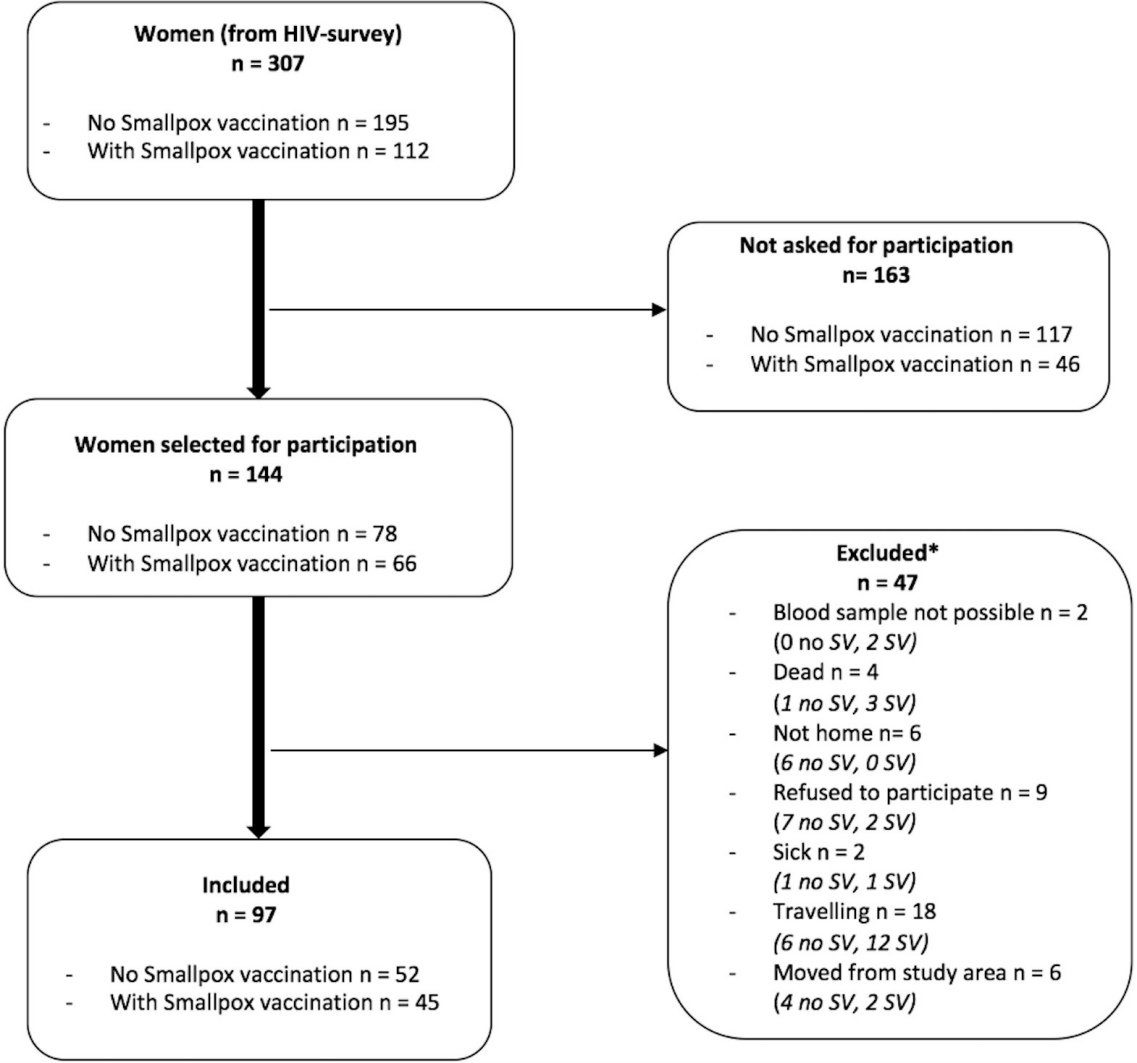
Flowchart of participants included in the study. *In parenthesis number of women with and without scar from smallpox vaccination in the given group. No SV = no smallpox vaccination, SV = smallpox vaccination.

All HIV-negative women participating in the survey and born before 1974 were eligible for inclusion into this study, which took place from 1 October to 25 October 2016; this amounted to 307 women (195 women without a smallpox vaccination scar and 112 women with a smallpox vaccination scar) (Fig 1).

Participants were enrolled into this study during two two-week periods. We invited as many women as possible to participate during these periods. On each day of inclusion, women who met the inclusion criteria were randomly selected, visited in their home, and asked for additional participation in this study. Each day we aimed to include an equal number of women with and without a smallpox vaccination scar. We managed to visit and invite a total of 144 women.

Only apparently healthy women were eligible. Women who had a fever or were feeling ill on the day of the visit were excluded from the study. The women were also excluded from the study if it was not possible to draw blood (difficult venepuncture or insufficient blood volume obtained), if the woman was not home, refused to participate, was travelling or had moved away from study area. Four women had died between the time of the HIV survey and sampling for this study. In total, 47 women were excluded from the study (Fig 1).

All participants filled in a short questionnaire and were subsequently offered a free consultation with a local doctor at the health centre nearby. At the consultation, the women were informed of the result of the HIV test.

### HIV diagnosis

All women included in the study were HIV seronegative in the previous HIVsurvey, and all women were HIV seronegative when retested in the current study. Screening for HIV was done using a rapid test (Determine HIV-1/2 assay, Abbott Laboratories).

### Assessment of vaccination status

There are no central registers for smallpox vaccination in Guinea-Bissau; assessment of vaccination status relied on the assessment of the characteristic smallpox vaccination scar similar to previous studies [7, 8, 11].

The prevalence of scars from smallpox vaccination, BCG vaccination and scars without a clear origin were assessed by a field worker trained in identifying and measuring vaccine scars. The height and width of the scar were measured with a ruler and noted on the questionnaire.

### Laboratory methods

Cryopreservation of cells causes down-regulation of CCR5 on the cell surface [15]. Antibody staining and analysis of surface CCR5 should therefore be performed on freshly isolated PBMCs. However, flow cytometry was not available in Guinea-Bissau during the study period. We therefore validated the following method:

Blood was sampled in special blood collection tubes containing a fixation liquid (TransFix, Caltag Medsystems ltd., Cytomark, Buckingham, UK) (Data and description of Transfix validation is available from figshare https://doi.org/10.6084/m9.figshare.7139687.v1).

### Flow cytometry

Blood samples were stained according to a T-cell maturation profile: FITC Mouse Anti-Human CD3 (clone UCHT1, cat. 561806, 2μL per test), PE-CF594 Mouse Anti-Human CD28 (clone CD28.2, cat. 562296, 2μL per test), PE Mouse anti-Human CCR7 (clone 150503, cat. 560765, 10μL per test), PerCP Cy5.5 Mouse Anti-Human CD8 (clone SK1, cat. 560662, 1μL per test), APC-H7 Mouse Anti-Human CD4 (clone RPA-T4, cat. 560158, 1.7μL per test), BV421 Mouse Anti-Human CCR5 (clone 3A9, cat. 565000, 5μL per test), BV605 Mouse Anti-Human CD27 (clone L128, cat. 562656, 1.7μL per test), BV786 Mouse Anti-Human CD45RA (clone HI100, cat. 563870, 5μL per test) (BD, Becton, Dickinson and Company, Franklin Lakes, New Jersey, U.S.). Antibodies were used in titrated amounts.

70 μL of whole blood was stained by addition of listed amounts of all antibodies in 50 μL BD Brilliant Stain Buffer. Incubation time was 30 minutes at room temperature in the dark.

**Fig 2:**
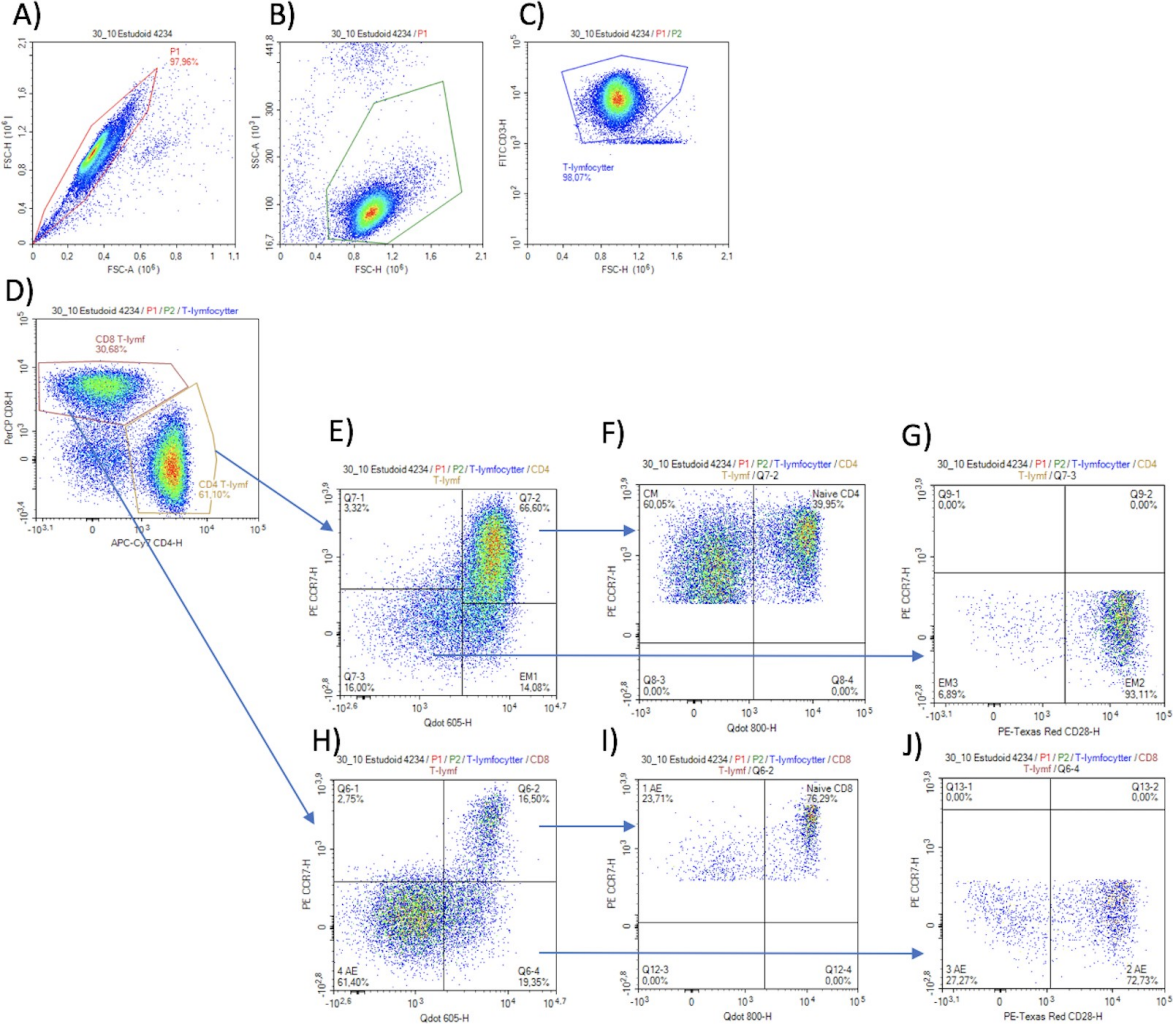
Plots of gating of CD4 and CD8 T-lymphocytes from flow cytometry. A) Singlets, B) lymphocytes, C) T-lymphocytes, D) CD4 and CD8 T-lymphocytes, E) Effector Memory 1 CD4, F) Central Memory and Naive CD4, G) Effector Memory 2 and Effector Memory 3, H) 4th Antigen Experienced, I) Naive CD8 and 1st Antigen Experienced, J) 2nd and 3rd Antigen Experienced.

The erythrocytes were then lysed by addition of 1.75 mL diluted BD FACS Lysing Solution (diluted 1:10 with sterile water) and incubated at room temperature in 20 minutes on a tilt board in the dark. The blood samples were then centrifuged at 300*g* for five minutes, the supernatant discarded, cells washed with 3 mL PBS and centrifuged again. Finally, the samples were analysed on a NovoCyte Flow Cytometer (ACEA Biosciences Inc., San Diego, CA, USA). A fluorescence threshold was used to limit analysis to CD3^+^ events (Fig 2C).

Flow cytometry data were gated and analysed with NovoExpress Software (ACEA Biosciences Inc., San Diego, CA, USA) by a researcher blinded to the vaccination status of the women.

#### Gating strategy (Table 1 and Fig 2)

Before analysis, all data were compensated for spectral overlap by running appropriate compensation controls (BD CompBeads lot. 4238681 cat. 552843) and using fully automated software compensation to derive the spectral overlap matrix and apply appropriate compensation. Data were compensated on the basis of median fluorescence (area) signals.

**Table 1:** Receptors of CD4 and CD8 T-lymphocyte subsets.

T-lymphocyte subset	Receptors
Naïve CD4	CD4^+^ CD27^+^ CCR7^+^ CD45RA^+^
Central Memory CD4	CD4^+^ CD27^+^ CCR7^+^ CD45RA^-^
Effector Memory 1 CD4	CD4^+^ CD27^+^ CCR7^-^
Effector Memory 2 CD4	CD4^+^ CD27^-^ CCR7^-^ CD28^+^
Effector Memory 3 CD4	CD4^+^ CD27^-^ CCR7^-^ CD28^-^
Naïve CD8	CD8^+^ CD27^+^ CCR7^+^ CD45RA^+^
1^st^ Antigen Experienced	CD8^+^ CD27^+^ CCR7^+^ CD45RA^-^
2^nd^ Antigen Experienced	CD8^+^ CD27^+^ CCR7^-^ CD28^+^
3^rd^ Antigen Experienced	CD8^+^ CD27^+^ CCR7^-^ CD28^-^
4^th^ Antigen Experienced	CD8^+^ CD27^-^ CCR7^-^

Singlet events were identified in a FSC-A versus a FSC-H plot (Fig 2A). A lymphocyte-gate was established on the basis of FSC-H and SSC-A (2B). T-lymphocytes were then defined as CD3 positive (2C) and further classified as either CD4 or CD8 T-lymphocytes (2D).

*Identification of CD4 T-lymphocyte subsets*; the CD4 positive T-lymphocytes were gated according to CD27 and CCR7, and the CD27 positive and CCR7 negative cells were labelled as Effector Memory 1 CD4 (2E). The CCR7 positive and CD27 positive cells were further gated according to CD45RA. In this plot, the CD45RA positive cells were labelled as Naïve CD4, and the CD45RA negative cells were labelled as Central Memory CD4 (2F). The CCR7 negative and CD27 negative cells were further gated using CD28. In this plot, the CD28 positive cells were labelled Effector Memory 2 CD4, and the CD28 negative cells were labelled as Effector Memory 3 (2G).

*Identification of CD8 T-lymphocyte subsets*; The CD8 positive T-lymphocytes were gated by CD27 and CCR7, and in this plot, the CD27 negative and CCR7 negative cells were labelled as 4^th^ Antigen Experienced (2H). From the previous plot, the CD27 positive and CCR7 positive cells were further gated according to CD45RA. The CD45RA positive cells were labelled Naïve CD8, and the CD45RA negative were labelled 1^st^ Antigen Experienced (2I).

The CD27 positive and CCR7 negative cells were further gated using CD28. The CD28 positive cells were labelled 2^nd^ Antigen Experienced and the CD28 negative cells were labelled 3^rd^ Antigen Experienced (2J).

### Statistics

#### Statistical analyses were performed using STATA/SE 13

Multivariable linear regression analyses were used to test for association between CCR5 expression both as a fraction and median fluorescence (dependent parameter) and scar (independent parameter). All analyses were adjusted for age and menstruation as the groups differed in these variables. A two-sided significance level of 5% was used.

In three of the subsets (Effector memory 3 CD4, 1 Antigen Experienced CD8 and 3 Antigen Experienced CD8), the data on CCR5 positive cell fraction was analysed as dichotomous (a cut off percentage was chosen to allow similarly sized groups) as the cell count was low in these subsets.

### Ethical consideration

Verbal and written information was given to the participating women before written consent was obtained. In illiterate individuals, a fingerprint was obtained. A local doctor and nurse informed the women about the result of the HIV-test, and treatment would have been offered if the HIV-test was positive. However, none of the women were tested positive of HIV.

The study was approved by the National Research Ethics Committee in Guinea-Bissau (No. Ref 012/CNES/INASA/2016) and received consultative approval from the National Research Ethics Committee of Denmark (Case No. 1606000).

## Results

### Study population

A total of 97 women were included in the study. Of these, 52 had a smallpox vaccination scar. Among the 97 women, 45 had a scar from BCG; the distribution of BCG scars did not differ between the women with and without a smallpox vaccination scar (p = 0.44).

The median age of the women was 51.6 years; women with a smallpox vaccination scar were, on average, 5.8 years older (*p<0*.*01*) than women without a scar.

No differences in Body Mass Index (BMI) or mid-upper arm circumference (MUAC) were found between the groups. There was a difference in the number of women who were still menstruating and women using contraception even after adjusting for age (*p = 0*.*001*) (Table 2).

**Table 2:** Characteristics of women with and without a smallpox vaccination scar (95% confidence Intervals).

	Smallpox vac (n = 52)	No smallpox vac (n = 45)	p-value
**Age (years)**	54.3 (52.1–56.5)	48.5 (46.7–50.3)	<0.01
Time until analysis (days)*	9.1 (8.4–9.8)	10.0 (9.3–10.7)	0.05
BMI**	27.6 (26.2–29.0)	27.5 (25.8–29.2)	0.95
**MUAC (cm)**	322 (309–336)	331 (314–348)	0.39
**BCG scar**	26 (50%)	19 (42%)	0.44
**Scar not identified as smallpox vac or BCG**	3 (5.8%)	1 (2.2%)	0.38
**Still menstruating**	8 (15.4%)	28 (62.2%)	<0.01
**Using hormonal contraception**	0	3 (6.7%)***	0.03

Vac = vaccination, BMI = Body Mass Index, MUAC = mid upper arm circumference, BCG = Bacille-Calmette-Guérin. Values on Age, Days until analysis, BMI and MUAC given as mean and 95% CI. Values on BCG, scar not identified as smallpox vaccination or BCG, Menstruation and use of contraception are given as quantity and percentage.

* Days from blood sampling in Guinea-Bissau to analysis at Aarhus University Hospital, Denmark

** Calculated as weight/(height*height). Weight in kilogram and height in meters

***2 used intrauterine device, 1 used hormonal contraception

No differences in ethnicity and educational level were found between the groups.

### CD4 and CD8 T-lymphocyte fractions

In the analysis of the blood samples using flow cytometry, no significant difference between vaccination groups was found in the fractions of CD4+ and CD8+ T-lymphocytes (p = 0.38 and p = 0.22).

### CCR5 positive cell fraction of CD4 and CD8 T-lymphocyte subsets

**Fig 3:**
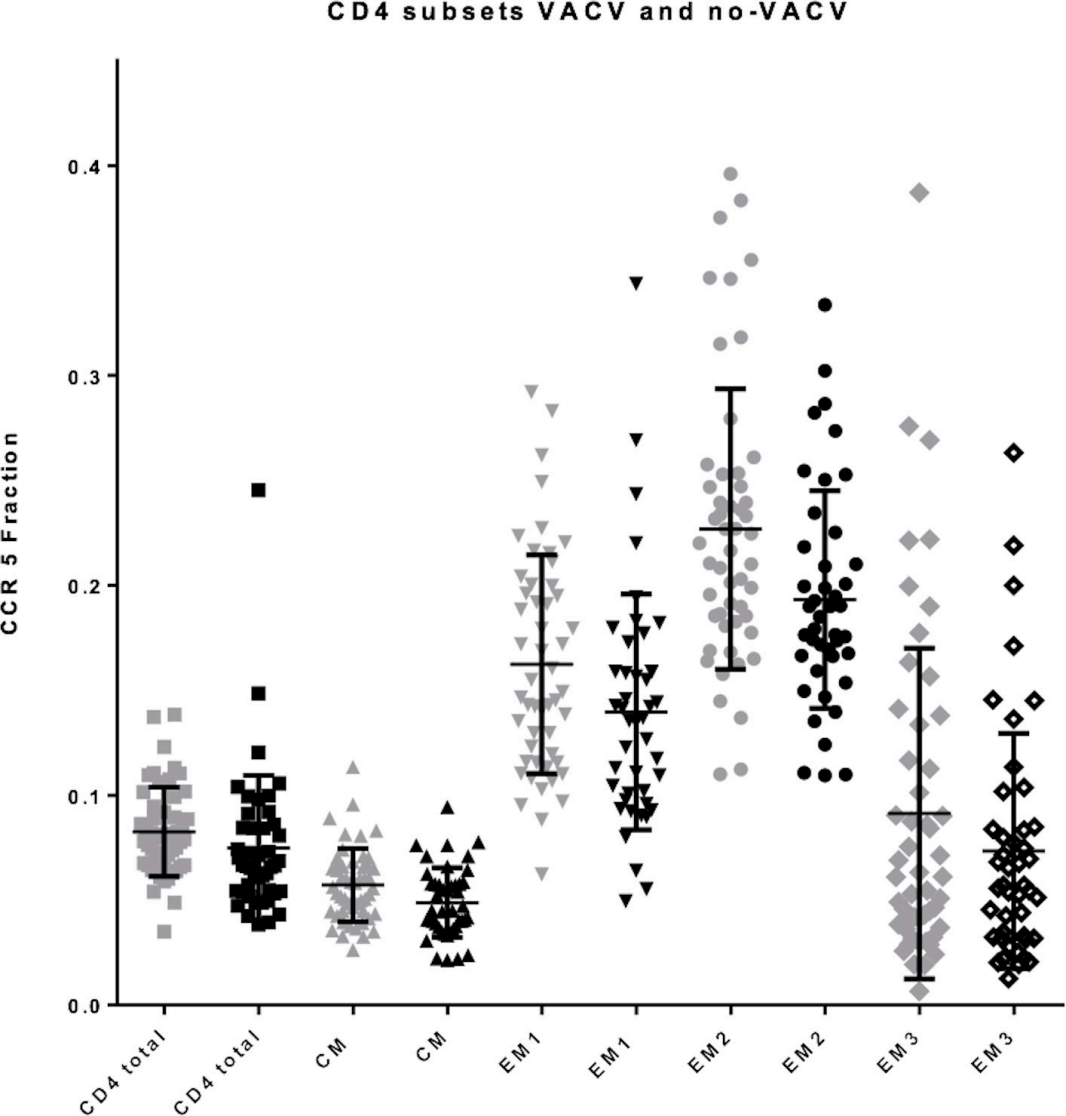
CCR5 positive cell fraction on CD4 subsets in women with and without a smallpox vaccination scar. Grey colour is smallpox vaccination and black colour is no smallpox vaccination. CCR5 positive cell fraction of CD4 subsets in the two groups. Mean with SD. Central Memory (CM), Effector Memory 1 (EM1), Effector Memory 2 (EM2), Effector Memory 3 (EM3).

**Fig 4:**
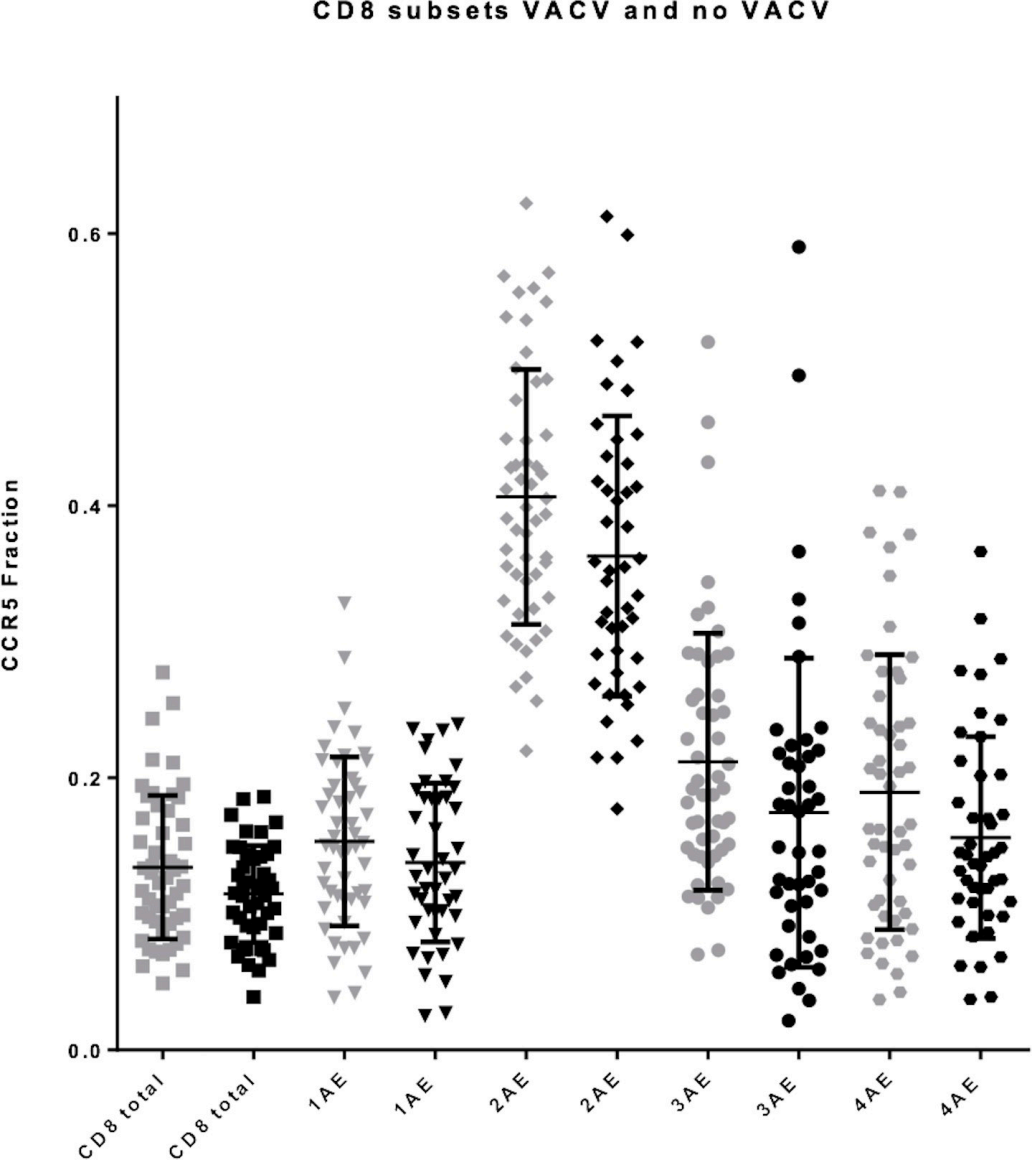
CCR5 positive cell fraction on CD8 subsets in smallpox vaccination group and no-smallpox vaccination group. Grey colour is smallpox vaccination and black colour is no smallpox vaccination. CCR5 positive cell fraction of CD8 subsets in the two groups. Mean with SD. 1. Antigen Experienced (1AE), 2. Antigen Experienced (2AE), 3. Antigen Experienced (3AE), 4. Antigen Experienced (4AE).

The subsets with the largest fraction of CCR5 positive cells were the CD28^+^ CCR7^-^ CD27^-^ CD4^+^ T-lymphocytes (Effector Memory 2) (Fig 3) and CD28^+^ CCR7^-^ CD27^+^ CD8^+^ (2^nd^ Antigen Experienced) (Fig 4).

Naïve CD4 and naïve CD8 T-lymphocytes were the subsets with the smallest fraction of CCR5 positive cells, with a median of less than 1% of cells expressing CCR5 (data available at online repository figshare https://doi.org/10.6084/m9.figshare.7139669).

In all T-lymphocyte subsets, the CCR5 positive cell fraction was usually highest among the women with a smallpox vaccination scar (Figs 3 and 4). However, no significant trend was found in any group after adjusting for age and menstruation status.

When analysing the median fluorescence of the anti-CCR5 staining of T-lymphocyte subsets, no difference was found between the groups of women with and without a smallpox vaccination scar (data available at online repository https://doi.org/10.6084/m9.figshare.7139720.v1).

In the three subsets (Effector memory 3 CD4, 1 Antigen Experienced CD8 and 3 Antigen Experienced CD8) where data on the CCR5 positive cell fraction was analysed as dichotomous (because the cell count was low in these subsets), we found no difference between the groups with respect to the risk of having a low or high CCR5 positive cell fraction.

As a difference in age was found between the groups, a possible correlation between age and CCR5 expression was investigated. No such correlation was found in any of the T-lymphocyte subsets in any group.

All included women had one scar from smallpox vaccination except four women who had two scars.

It was analyzed if size of the smallpox vaccination scar was associated with the CCR5 positive cell fraction in all T-lymphocyte subsets. The size of the scar was analyzed as a continuous variable and the women with a scar from smallpox vaccination were divided into three groups according to the size of the scar. There was no significant association between size of the scar and the CCR5 expression in any T- lymphocyte subset.

## Discussion

Epidemiological studies have found that smallpox vaccination may have non-targeted beneficial effects on survival [7–10] and mediates a protective effect against HIV-1 in women [11]. However, little is known about the immunological mechanisms behind this protective effect.

To date, studies on the immunological protective effects of smallpox vaccination against HIV-1 have been performed *in vitro/ex vivo* and have investigated the effect of smallpox vaccination days and months after infection [12, 13]. To our knowledge, our study is the first to investigate the *in vivo* effect of smallpox vaccination on CCR5 expression in women.

The study was inherently only able to investigate long-term effects of smallpox vaccination.

Analysing the blood samples from 52 women with a smallpox vaccination scar and 45 without a smallpox vaccination scar, we did not find any significant differences in the expression and quantity of the CCR5 receptor between the groups. Thus, the results do not support the hypothesis that down-regulation of the CCR5-receptor can explain a long-term protective effect against HIV-1.

### Strengths and weaknesses

Social differences between vaccinated and non-vaccinated women are possible. The women were vaccinated against smallpox in childhood, and we expect that the women who were vaccinated during the time of out-phasing of the smallpox vaccination program were predominantly vaccinated by coincidence and not because of general health or social differences. However, in the smallpox mortality study in Guinea-Bissau from 1998–2002 [7], the prevalence of smallpox vaccination scars differed by ethnic group, number of rooms and type of roof in connection with housing, but not by educational level and place of birth.

In the current study, no difference was found in educational level and ethnicity between vaccinated and non-vaccinated women.

Some of the challenges associated with research in a low-resource country are related to cultural differences. In Guinea-Bissau, the study participants did not always know their exact age. However, we find it unlikely that this will introduce a bias in one group and not the other. In addition, there is a risk of misclassification of the scars from BCG, smallpox vaccination or those identified as neither. However, the field worker was trained in reading the scars and the risk of misclassification of scars is not believed to be large in any of the groups. It would however strengthen this study by complementing with a substudy with assessment of vaccinia-specific immune responses in the two groups of women.

**Fig 5:**
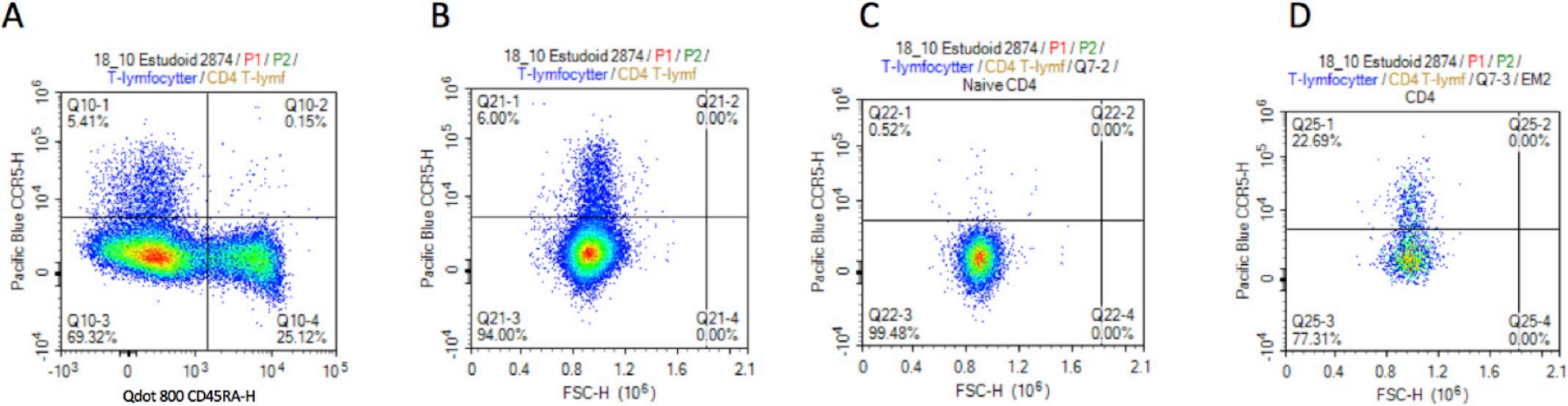
Plots of CD4 T-lymphocytes from flow cytometry. A) CD4 T-lymphocytes gated by CD45RA and CCR5, B) CD4 T-lymphocytes gated by FSC-H and CCR5, C) Naïve CD4 T-lymphocytes gated by FSC-H and CCR5, D) Effector Memory 2 CD4 T-lymphocytes gated by FSC-H and CCR5.

**Fig 6:**
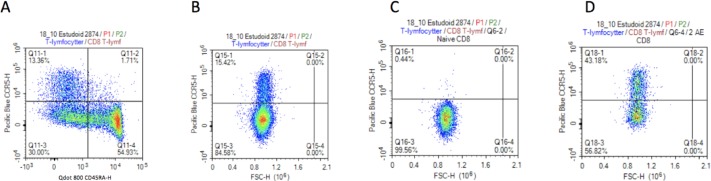
Plots of CD8 T-lymphocytes from flow cytometry. A) CD8 T-lymphocytes gated by CD45RA and CCR5, B) CD8 T-lymphocytes gated by FSC-H and CCR5, C) Naïve CD8 T-lymphocytes gated by FSC-H and CCR5, D)2. Antigen Experienced CD8 T-lymphocytes gated by FSC-H and CCR5.

The classification of CCR5 expressing and non-expressing T-cells is demanding. The few studies that show the gating strategy display a similar image of the CCR5 population as not clearly separated in a negative and positive population (Figs 5 and 6) [16, 17]. Furthermore, the finding that the largest fraction of CCR5 positive cells on Effector Memory 2 and 2^nd^ Antigen Experienced is in line with current literature on the topic [18] (Figs 3 and 4).

Transfix has previously been used for the stabilisation of whole blood for delayed analysis up to fourteen days [19–21]. It is possible that this method is source of error as the separation between CCR5 positive and negative populations might decrease over time, and hereby the classification of the CCR5-positive T-cells will be even more demanding. The risk of misclassification of CCR5-positive and -negative cells is however not believed to be bigger in any of the groups.

Our study population was defined by including women who had survived to a certain age and were HIV-1 negative. As we know that smallpox vaccination is associated with lower mortality [7–10] and a tendency of a lower risk of HIV-1 among women in Guinea-Bissau [11], it is possible that our results are affected by selection bias, if more fragile and HIV-1 positive women in the control group had died before we conducted our study.

The epidemiological studies from Guinea-Bissau found mainly a protective effect against HIV-1 among individuals with two or more vaccinia scars [11]. Number of scars was also important for the beneficial effect on survival [7, 8]. In the present study, only 4 individuals had two or more scars so it was not possible to examine whether number of scars had an effect on CCR5 expression. In the epidemiological studies [7, 8, 11] the protective effect of vaccinia was found in relation to individuals who had received neither vaccinia nor BCG. Given the limited size of the present study, there was insufficient power to compare with the group which had had received neither vaccinia nor BCG.

### Consistency with other studies

Our results contrast findings of other studies investigating the short-term effect of smallpox vaccination against HIV-1 *in vitro/ex vivo* [12, 13]. Specifically, *Vanpouille et al* [12] reported that smallpox vaccination predominantly depletes CCR5-positive T-lymphocytes compared with CCR5-negative T-lymphocytes in tonsillar tissues infected by smallpox vaccination *ex vivo*. They also found that smallpox vaccination significantly inhibits replication of the HIV-1 strain using CCR5 as a co-receptor in human tonsillar tissues co-infected *ex vivo* with smallpox vaccination and HIV-1, when these results are compared with human tonsillar tissues only infected with HIV-1.

HIV-1 susceptibility in PBMCs from individuals immunized with the vaccinia virus in the preceding three-six months was compared with those from vaccinia naïve donors in the study by *Weinstein et al* [13]. Vaccinia immunization results in an up to five-fold reduction in the CCR5-tropic but not the CXCR4-tropic HIV-1 replication in the cells from vaccinated individuals. This effect is not mediated by a release of the chemokines MIP-1α, MIP-1β and RANTES; an alteration in CCR5 is suggested to explain this.

It would thus be very interesting in further studies to explore the long-term effect of smallpox vaccination by investigating HIV-1 susceptibility and inhibition of replication of HIV-1 in PBMCs from individuals who have been immunized with vaccinia in childhood.

### Interpretation

Sexually transmitted diseases (STD) increase the risk of HIV-acquisition [22] and might interact with the proposed effect of smallpox vaccination. It could be hypothesised that smallpox vaccination induces a down-regulation of the CCR5-receptor on previously activated cells during an STDinfection and thereby induces a protection against HIV-1 infection. In our study of healthy women, we found relatively few activated cells. As CCR5 expression is increased on activated cells [23–26], we cannot exclude that results could be different in individuals with more pronounced T-cell activation.

Our study is limited by only considering cells in peripheral blood, whereas other, less accessible compartments may have a greater impact on HIV susceptibility.

As the protective effect of smallpox vaccination against HIV-1 has been found to be greater in women than men, it could be argued that lymphocytes associated with the vaginal mucosa may be more relevant. This idea is supported by the observation that the protective effect is not seen among injection drug users [11].

Both smallpox and BCG vaccinations are administered subcutaneously, and studies in mice have shown that memory acquired at the site of vaccination against smallpox spread throughout the entire epithelial surface [27]. Thus, enhanced epithelial protection would be particularly protective against vaginally acquired HIV-1 infections. It would be interesting to investigate a down-regulation of the CCR5-receptor in T-cells in the vaginal mucosa.

## Conclusion

Smallpox vaccination was not associated with a long-term down-regulation of the CCR5 receptor on the surface of peripheral T-lymphocytes among HIV-negative women.

This study does not support the theory of long-term down-regulation of the CCR5-receptor on PBMC’s as an explanation for the lower HIV-1 susceptibility among smallpox vaccinated women.
